# Progressive multifocal leukoencephalopathy (PML) following autologous peripheral blood stem cell transplantation for multiple myeloma

**DOI:** 10.1002/ccr3.2612

**Published:** 2020-03-20

**Authors:** Karina M. Bennett, Neill Storrar, Peter Johnson, Peter M. Fernandes

**Affiliations:** ^1^ Department of Neurology NHS Lothian Western General Hospital Edinburgh UK; ^2^ Department of Haematology NHS Lothian Western General Hospital Edinburgh UK; ^3^ Centre for Clinical Brain Sciences University of Edinburgh Edinburgh UK

**Keywords:** autologous peripheral blood stem cell transplantation, multiple myeloma, progressive multifocal leukoencephalopathy

## Abstract

PML should be considered in patients with neurological symptoms following MM and in those who are immunosuppressed. Symptoms are diverse and often rapidly progressing. Prompt referral and early involvement of the multidisciplinary team are crucial.

## Graphical abstract

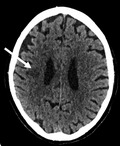

## 1 INTRODUCTION

A 67‐year‐old man presented with left‐sided weakness and reduced left‐hand dexterity four months after autologous stem cell transplant (ASCT) for multiple myeloma (MM). Clinical neurological examination was felt to be normal. Three months later, he reported progressive worsening of this weakness; neurological examination showed mild left‐sided facial asymmetry.

He was diagnosed with IgG lambda MM in March 2016 during follow‐up of lytic lesions on a chest radiograph performed because of recurrent chest infections. His past medical history included chronic obstructive pulmonary disease and controlled hypertension. He was *human immunodeficiency virus* (HIV) negative. He underwent induction treatment with four cycles of bortezomib, thalidomide, and dexamethasone starting in April 2016, complicated by neutropenic sepsis requiring intensive care for high‐flow oxygen and granulocyte‐colony stimulating factor. He also developed thrombosis of his peripherally inserted central catheter, treated with low molecular weight heparin. ASCT with melphalan conditioning (200 mg/m^2^) was carried out in January 2017. On day 7 following ASCT, he developed neutropenic sepsis from urinary *Escherichia coli*. He took routine post‐transplant *Pneumocystis jirovecii* prophylaxis trimethoprim/sulfamethoxazole, which was switched to pentamidine in April 2017 because of slow platelet count recovery. This stopped in July 2017. In March 2017, he had an episode of herpes zoster affecting the left trunk.

General weakness is common in the months following transplant as the patient recovers from the intensive treatment, complications and hospital admission. The focal progressive symptoms and new findings on neurological examination are much more concerning. There is a wide differential diagnosis, including cerebrovascular disease, demyelinating illness and space occupying lesions.

**Figure 1:**
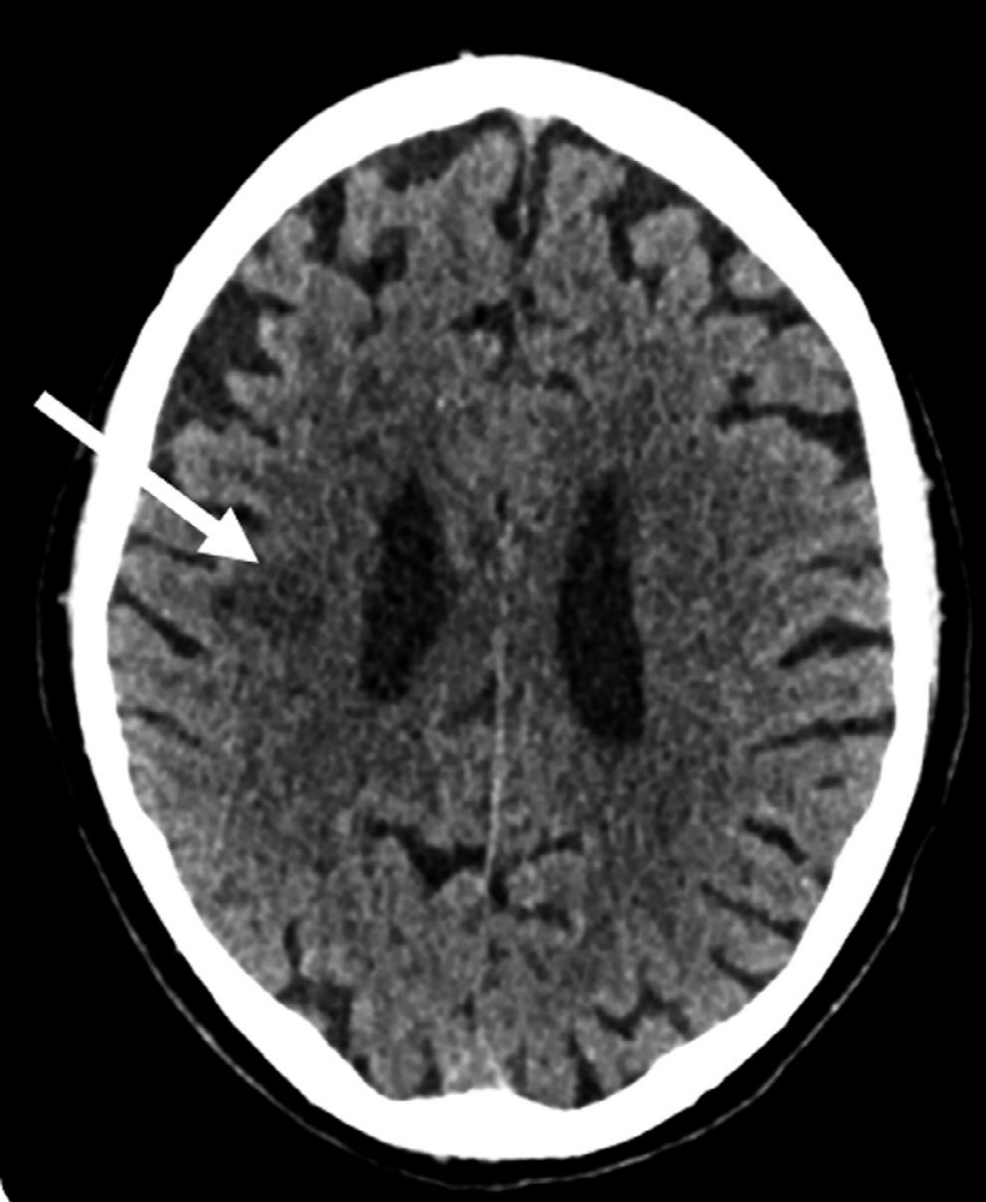
Computerized tomography (CT) scan of the head was performed which showed multiple areas of low attenuation, as shown by the white arrow

Computerized tomography (CT) scan of the head was performed which showed multiple areas of low attenuation (Figure 1).

The radiological findings were thought to represent multiple areas of infarction, likely embolic in nature. The progressive nature of the weakness could represent multiple infarcts over time. The patient had several risk factors for stroke: smoking history, hypertension, malignancy, and treatment with chemotherapy. 1 The complication of peripherally inserted central catheter thrombosis could also point toward a predisposition for thrombosis.

**Figure 2:**
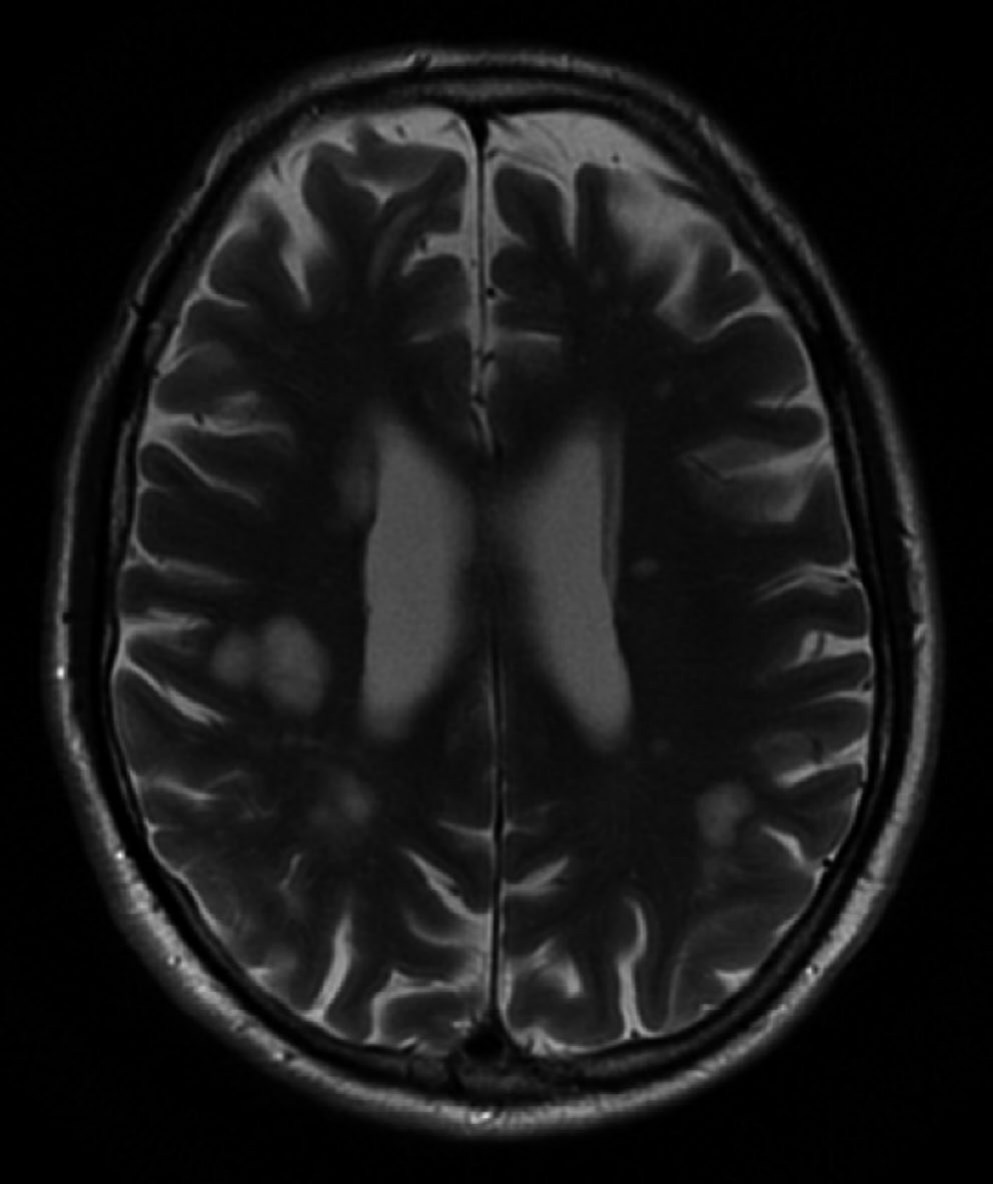
Magnetic resonance imaging of the brain showed numerous hyper‐intense T2 lesions within the subcortical white matter of both cerebral hemispheres

**Figure 3:**
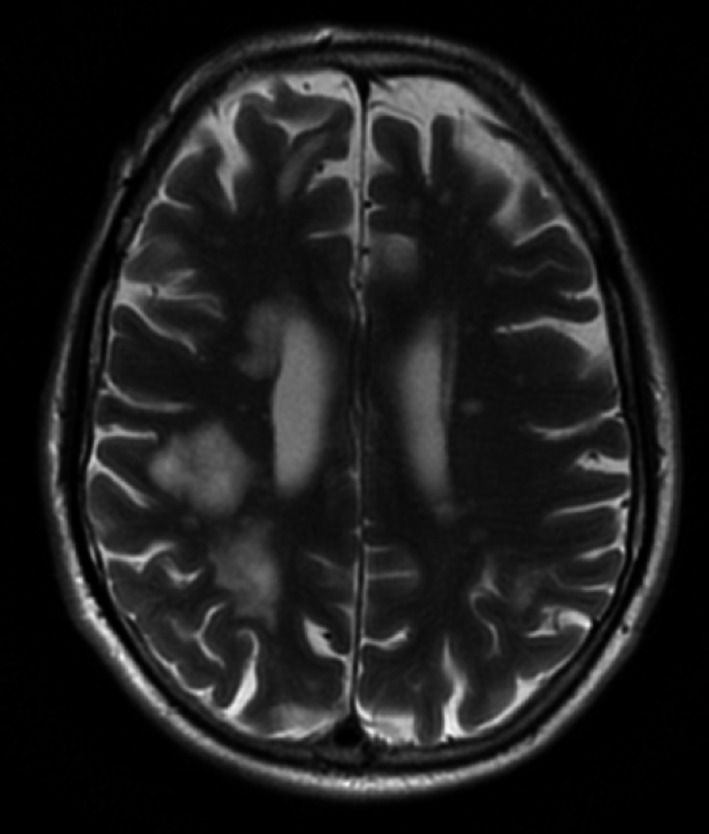
Magnetic resonance imaging (MRI) repeated one month after the MRI shown in Figure 1 shows interval progression in lesion size

Magnetic Resonance Imaging (MRI) of the brain showed numerous hyper‐intense T2 lesions within the subcortical white matter of both cerebral hemispheres (Figure 2). The MRI was repeated one month later and showed interval progression in lesion size (Figure 3).

The MRI head showed multiple areas T2 high signal in the white matter, in keeping with demyelination. This is a feature of several neurological conditions, including hypoxic/ ischemic insults, inflammation (multiple sclerosis, acute disseminated encephalomyelitis, vasculitis, and sarcoidosis), metabolic/toxic causes (carbon monoxide poisoning, vitamin B12 deficiency, central pontine myelinolysis, inherited leukodystrophies), and infections (HIV, syphilis, lyme disease, and progressive multifocal leukoencephalopathy (PML)).

His clinical symptoms continued to progress, and by September 2017, he was unable to walk 2 m unaided. He developed emotional lability and motion sickness. There was no alteration in sensation, sphincter or bulbar symptoms, and he had no weight loss, night sweats or fevers.

Examination showed normal tone bilaterally. There was mild left‐sided weakness of the face and left‐sided hemiparesis, worse than previous. Reflexes in the legs were brisk with some spreading, and the left plantar response was up‐going. Gait was narrow‐based, stiff and hemiplegic. There were no cerebellar signs. Sensation was intact. Assessment of cognitive function with Addenbrooke's Cognitive Examination III‐revised (ACE III‐R) showed mild cognitive impairment with a score of 84/100 (attention 17/18, memory 21/26, fluency 7/14, language 26/26, and visuospatial 13/16).

Interpretation of ACE III‐R scores is difficult, and repeated tests at different points in time are the best way to show cognitive decline. A score of less than 88 gives a significant likelihood of dementia 2; thus, in our patient, a score of 84 demonstrates probable cognitive impairment.

Further investigations were performed to determine the cause of the demyelination. A CT chest, abdomen, and pelvis showed no evidence of systemic disease, such as solid malignancy. Serum serology was negative for HIV, syphilis, and lyme disease. Connective tissue autoimmune screen was negative, with no evidence of systemic vasculitis, and vitamin B12 levels were normal. A lumbar puncture showed normal cell count (red blood cell 0 × 10^12^/L, white blood cell 2 × 10^9^/L), protein (0.37 mg/dL), glucose (3.4 mg/dL), and no oligoclonal bands in serum or cerebrospinal fluid (CSF). Viral polymerase chain reaction was negative for enteroviruses, herpes simplex virus 1 & 2, parechovirus, and varicella zoster virus, and there were no abnormal cells on microscopy. *John Cunningham polyomavirus virus* (JCPyV), the causative agent of PML, was positive in serum (1.55 index value); CSF testing for JCPyV was strongly positive at a value of 830 000 copies/ml.

Seropositivity for JCPyV in this patient was not unexpected as between 38% and 82% of the normal population have positive serology, but the virus should not be present in the CSF.^3^, ^4^ The positive CSF for JCPyV with compatible clinical and radiological findings fulfill the diagnostic criteria for PML,^5^ and there was no diagnostic need to perform a brain biopsy. This diagnosis was unexpected as the patient did not appear to be significantly immunocompromised following ASCT and PML is rare in this patient group.

Key blood results before and after ASCT are shown in Table 1, and further measures of immune functioning were taken after the diagnosis of PML to evaluate scope for optimization of immune function (Table 2).

**Table 1:** Table showing key blood results before, immediately after and 1, 2, and 3 months after ASCT

Time	IgG lambda paraprotein (g/L)	IgA (g/L)	IgM (g/L)	Hb (135‐180 g/L)	WCC (4.0‐11 10^9^/L)	Lymphocytes (1.5‐4 10^9^/L)	Neutrophils (2.0‐7.5 10^9^/L)	Platelets (150‐400 10^9^/L)
Diagnosed MM (March 2016)	24	0.36	0.14	‐	‐	‐	‐	‐
Before chemotherapy (April 2016)	16			130	14.6	0.23	14.09	143
Immediately post ASCT (January 2017)	<1	1.25	0.20	117	5.2	0.13	4.85	141
1 month (February 2017)	<1			95	5.2	0.85	3.19	39
2 months (March 2017)	<1			92	6.7	0.52	5.47	100
3 months (April 2017)	<1	0.87	0.13	98	5.6	0.67	4.3	57

**Table 2:** Measures of immune functioning at the time of diagnosis of PML

Immune measure	Result
Lymphocytes (1.5‐4 cells 10^9^/L)	0.41
B cells (10%‐16%)	43
T‐cells (67%‐76%)	40
CD4 absolute (500‐1500 cells u/L)	72
CD4% (29%‐61%)	18
CD8 absolute (250‐750 cells u/L)	90
CD8% (14%‐44%)	22
CD4/CD8 ratio (0.9‐3.5)	0.8
Natural killer cells (10%‐15%)	16
C3 (0.81‐1.57 g/L)	1.44
C4 (0.13‐1.39 g/L)	0.34
IgA (6‐15 g/L)	0.58
IgG (0.8‐4.5 g/L)	6.82
IgM (0.35‐2.9 g/L)	0.29

ASCT improves the duration of remission and overall survival in patients with MM although most patients develop relapse of MM after a period of remission. 6, 7, 8 The recovery of different immune cells occurs at different time points post‐transplant. The reconstitution of innate immunity occurs rapidly, within 20‐30 days, while reconstitution of adaptive immunity can take 1 year. 9 Early recovery of absolute lymphocyte count (ALC) can be used as a prognostic indicator of overall survival and progression free survival. 10 The patient followed the usual pace of immune reconstitution, with neutrophil recovery at day + 12; implying good immune functioning. Reactivation of *varicella zoster virus* is common after ASCT 11 and does not suggest unusual immunodeficiency for this patient group. The CD4 count is low; in cases of HIV‐associated PML, there appears to be an association between CD4 count and PML. 5

Treatment for JCPyV was commenced with mirtazapine 30mg once daily and intensive physiotherapy.

Treatment for PML can be tackled in two ways: restoring the immune function and attacking the virus. Restoring the immune functioning in the patient was problematic as he did not appear to have reversible immune suppression beyond normal recovery post‐ASCT. No methods to restore immune function were thought feasible except for the possibility of performing a second ASCT using remaining stem cells cryopreserved prior to the first ASCT. Several experimental antiviral therapies were considered, including mirtazapine, which is thought to block virus entry into glial cells via 5HT2a serotonin receptors. 12 Recently, three groups have reported favorably on the use of immune‐checkpoint inhibitors to treat PML; immunotherapy appears promising but requires further study. 13, 14, 15

During admission, his condition continued to worsen. He developed hospital acquired pneumonia (HAP) and deteriorated rapidly, dying 5 days later. His death was 18 months after diagnosis of MM, 10 months after ASCT, 14 days after diagnosis of PML and 10 days after mirtazapine had started. His death was before the second ASCT could be attempted.

It was very difficult to give a prognosis to the patient due to lack of comparable cases. His rapid deterioration from HAP was unexpected. This could reflect a weaker immune function than suggested by his blood test results. It was unlikely that a second stem cell infusion (without conditioning chemotherapy) would have promoted immune reconstitution, but equally seemed unlikely to cause harm.

## 2 DISCUSSION

PML is a rare demyelinating disorder of the central nervous system caused by reactivation of JCPyV virus, a neurotropic polyomavirus.^16^ The clinical presentation depends on the extent of demyelination and brain structures involved. Symptoms include muscle weakness, sensory deficit, hemianopia, cognitive dysfunction, aphasia, and coordination and gait difficulties. 17 JCPyV virus is present in 38%‐82% of the population 3, 4 but is usually well‐controlled by the immune system, resulting in asymptomatic latent infection. The kidney and bone marrow are the likely sites of latent infection,^18^ and there should be no JCPyV virus detectable in the CSF. PML primarily affects those with chronic and severely suppressed immune systems. 19 Three associations account for 90% of cases: (1) HIV; (2) immunosuppressing hematological malignancies, and (3) Multiple sclerosis patients treated with natalizumab, a monoclonal antibody to alpha‐4 integrin that prevents immune cells accessing the central nervous system. 20 PML is rarely associated with organ transplantation, solid malignancies, sarcoidosis, and autoimmune disorders.^20^

Treatment for PML is via restoring immune function and/or attacking the virus. Restoring immune function can be achieved by reversal of the immunosuppression caused by a disease, for example antiretroviral drugs in HIV 21; removal of immunosuppressive treatment for example stopping Natalizumab 22 or speeding drug clearance through plasma exchange. 23 The second option is to attack the virus. Pavlovic et al 20 conducted a review of treatment options and divided them into 3 main groups: (1) antiviral agents (JCPyV cell entry inhibitors, retrograde transport inhibitors, inhibitors of DNA replication, antimalarials, poly‐ADP‐ribose polymerase‐1 inhibitors and tyrosine kinase inhibitors), (2) immune response modulators (cytokines and inflammation inhibitors), and (3) immunisation strategies (passive and active immunisation). Research supporting these options is limited to case studies and retrospective studies with no strong consensus. Identification of successful treatment is difficult due to lack of an adequate animal model, small patient numbers, and rapid disease progression.^20^

Prognosis in PML is poor. In HIV, 70% are alive at one year and 50% at 2 years. In those with MS treated with Natalizumab, 77% are alive at three years, and in those with active immunosuppressing hematological malignancies, 10% are alive at 2 months. 20 A younger age at diagnosis, lower viral load of JCPyV, milder functional disability prior to PML diagnosis and more localized MRI brain involvement all appear to predict survival. 24

Developing PML after MM is rare with 11 case reports in the literature. 25, 26, 27, 28, 29, 30, 31, 32, 33, 34, 35 Presentations varied in these patients depending on the location of the lesion and included: hemiparesis and hemiplegia, 25, 28, 29, 32, 35 numbness and tingling of the legs,^29^ rapid cognitive decline, 25, 27, 33 homonymous hemianopia, 27 ataxia, 27 speech difficulties, 26, 30, 31, 32, 35 incontinence, 26 apathy, 29 and memory impairment. 32, 33, 34 ^.^ Trying to identify if an individual aspect of the MM treatment was responsible for the development of PML is difficult from these isolated case studies. Thalidomide has indirectly been related in this case and two others 29, 30 and lenalidomide in four cases. 25, 26, 27, 28 ASCT was used in all of the cases except two in which lenalidomide was used alone. 25, 28 Lenalidomide is an immunomodulatory drug with antiangiogenetic, anti‐inflammatory, and antiproliferative effects. Ying (2017) completed a systematic review and meta‐analysis of the risk of serious infection in patients with MM receiving lenalidomide and found that the overall incidence of high‐grade infection was 14.32%. Treatment for the PML varied in these case reports. Mirtazepine was used as a single agent in this case and 2 others; 26, 27 in these cases, the patients died within 2‐4 months. Yokokawa (2016) used mirtazepine in combination with mefloquine. In this case, the patient survived for more than one year from PML onset. Ripelino (2011) used mirtazapine in combination with cidofovir and the patient survived for more than two years after onset of PML. It is not possible to recommend the most appropriate therapy from these reports; however, the use of combined treatments may improve outcome. Treatment outcome may also be improved with early diagnosis.

There are difficult ethical questions about exposing patients to potentially harmful treatments when there is a lack of evidence of efficacy, even when prognosis is poor. Furthermore, any drug which is used must be able to cross the blood brain barrier and have acceptable side effects. There also needs to be consensus on how to measure outcome. Some possible measures include: survival, neurological examination, radiological lesion load and changes in JCPyV in the CSF. Until randomized control trials become available, the treatment of PML relies on case descriptions, retrospective studies, and informed discussions with patients.

PML is a rare complication of MM therapy and is associated with high mortality and morbidity. Clinicians should consider this diagnosis when assessing patients with neurological symptoms and signs following treatment for MM. It is important that referrals are made promptly, and there is involvement of the multidisciplinary team with haematologists, neurologists, radiologists, virologists, and therapists.

## CONFLICT OF INTEREST

None declared.

## AUTHOR CONTRIBUTION

KB, NS, PJ, and PMF: substantially contributed to conception and design, acquisition of data, or analysis and interpretation of data; drafted the article or revised it critically for important intellectual content; and finally approved the version to be published. Karina Bennett will act as guarantor for the content.
